# Remarkably High Mobility Thin-Film Transistor on Flexible Substrate by Novel Passivation Material

**DOI:** 10.1038/s41598-017-01231-3

**Published:** 2017-04-25

**Authors:** Cheng Wei Shih, Albert Chin

**Affiliations:** Department of Electronics Engineering, National Chiao Tung University, Hsinchu, 300 Taiwan

## Abstract

High mobility thin-film transistor (TFT) is crucial for future high resolution and fast response flexible display. Remarkably high performance TFT, made at room temperature on flexible substrate, is achieved with record high field-effect mobility (*μ*_*FE*_) of 345 cm^2^/Vs, small sub-threshold slope (*SS*) of 103 mV/dec, high on-current/off-current (*I*_*ON*_/*I*_*OFF*_) of 7 × 10^6^, and a low drain-voltage (V_D_) of 2 V for low power operation. The achieved mobility is the best reported data among flexible electronic devices, which is reached by novel HfLaO passivation material on nano-crystalline zinc-oxide (ZnO) TFT to improve both *I*_*ON*_ and *I*_*OFF*_. From X-ray photoelectron spectroscopy (XPS) analysis, the non-passivated device has high OH-bonding intensity in nano-crystalline ZnO, which damage the crystallinity, create charged scattering centers, and form potential barriers to degrade mobility.

## Introduction

The flexible electronics is the key technology for bendable light weight display^1–7^. Similar to traditional rigid displays, the higher mobility TFT^1–24^ is demanded for next generation higher density, faster speed, and lower power flexible display^6^. The amorphous InZnGaO (IGZO) TFT^5–10^ is a potential candidate for flexible electronics due to the low off-current (*I*_*OFF*_) from its large energy bandgap and high mobility of overlapped s-orbitals. However, the major issue for IGZO TFT is the rare Indium quantity in Earth’s crust and its high price. Alternatively, crystallized ZnO TFT is another candidate due to its better crystallinity than amorphous structure, similar with the much higher mobility of poly-Si than amorphous-Si TFTs. However, no high mobility poly-ZnO TFT was reported to date. In this paper, we demonstrate a high performance nano-crystallized ZnO TFT on low-cost Polyethylene naphthalate (PEN) flexible substrate processed at room temperature. Remarkably high *μ*_*FE*_ of 345 cm^2^/Vs was achieved in nano-crystalline ZnO TFT on flexible substrate with a high dielectric constant (high-κ) gate oxide^25–29^. This *μ*_*FE*_ value is even higher than IGZO and ZnON TFTs made on rigid glass^22, 23^ that is also the record highest value for devices on flexible substrate^1–7^. The high mobility is further supported by the low interface trap density from the small *SS* of only 103 mV/dec. Besides, high *I*_*ON*_/*I*_*OFF*_ of 7 × 10^6^ and a low V_D_ of 2 V were measured to reach low switching power of ${C}_{G}{V}_{D}^{2}f / 2$, where *C*_*G*_ and *f* are the gate capacitance and operation frequency, respectively. This remarkably high mobility TFT is achieved using novel HfLaO passivation on nano-crystalline ZnO. For comparison, the control non-passivated device has a *μ*_*FE*_ of 43 cm^2^/Vs. To understand such large mobility improvement, the X-ray photoelectron spectroscopy (XPS) was performed. The non-passivated ZnO showed a strong OH bonding signal. The formed HO-Zn-OH compound via moisture absorption will break the Zn-O bonding in ZnO crystal to form dangling bonds and charged scattering centers to lower the mobility strongly. In contrast to other passivation methods^14–21^, the LaO-based dielectric has important advantage of moisture absorption to lower the OH bonding formation as evident from the XPS data^25–29^. The remarkably high *μ*_*FE*_ HfLaO/ZnO TFT suggests the excellent opportunity for both flexible and rigid display applications.

## Results

**Figure 1:**
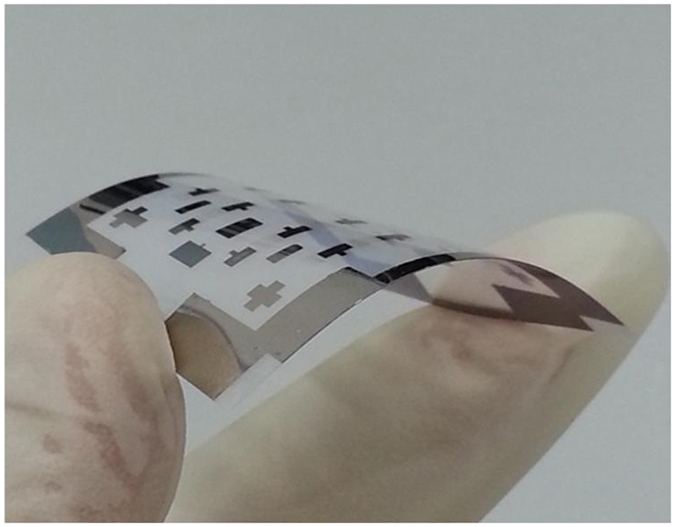
Device photo of fabricated TFTs on flexible PEN substrate.

The fabricated devices on flexible PEN substrate is shown in the photo of Fig. 1. Figure S1(a,b) show the capacitance-voltage (*C*-*V*) and current-voltage (*J*-*V*) characteristics of Al/high-κ metal-gate capacitor fabricated on the same flexible PEN substrate, respectively. A high capacitance density of 0.35 μF/cm^2^ was measured that lead to an equivalent-oxide-thickness (EOT) of 9.9 nm. A still low leakage current of 1.4 × 10^−5^ A/cm^2^ was measured at 1.5 V, even processed at room temperature. This is due to the merit of high-κ dielectrics, especially the higher κ TiO_2_ dielectric. The TiO_2_ has higher κ value for low voltage operation. To improve the interface^30^, extra SiO_2_ dielectric was inserted between ZnO and TiO_2_. To improve the leakage current via the low conduction band offset (ΔE_C_) of TiO_2_, stacked TiO_2_/HfO_2_ were applied^31^.

**Figure 2:**
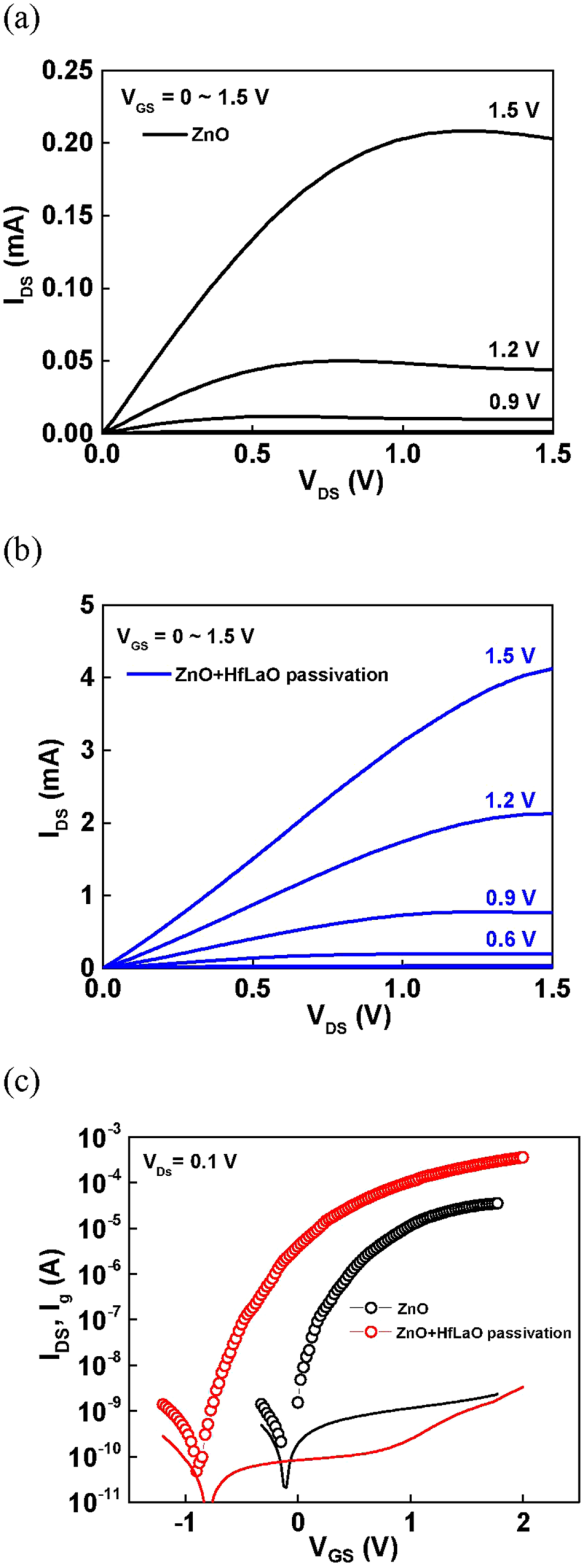
*I*_*DS*_-*V*_*DS*_ and characteristics of ZnO/high-κ/TaN TFT on flexible PEN (**a**) without and (**b**) with HfLaO passivation. (**c**) *I*_*DS*_-*V*_*GS*_ characteristics of ZnO/high-κ/TaN TFT on flexible PEN without and with HfLaO passivation.

Figure 2(a–c) show the transistor’s drain-source current versus drain-source voltage (*I*_*DS*_-*V*_*DS*_) and *I*_*DS*_ versus gate-source voltage (*I*_*DS*_-*V*_*GS*_) characteristics of ZnO/high-κ/metal-gate TFTs with and without HfLaO passivation. The ZnO TFTs without passivation show reasonable performance of *I*_*ON*_/*I*_*OFF*_ of 2 × 10^5^, *SS* of 112 mV/dec, and a V_T_ of 0.78 V. Here the gate leakage current is lower than *I*_*OFF*_ due to the thick stacked gate dielectric. The ZnO TFTs after HfLaO passivation shows more than one order of magnitude higher *I*_*ON*_ and 4 times lower *I*_*OFF*_, with large *I*_*ON*_/*I*_*OFF*_ of 7 × 10^6^, small *SS* of 103 mV/dec, and a low V_T_ of 0.13 V. These good device integrities were achieved at a low V_D_ of 2 V that is crucial to lower the switching power by orders of magnitude than existing TFT devices. Besides, the steep *SS* can also turn on the transistor faster for a lower voltage and power operation.

**Figure 3:**
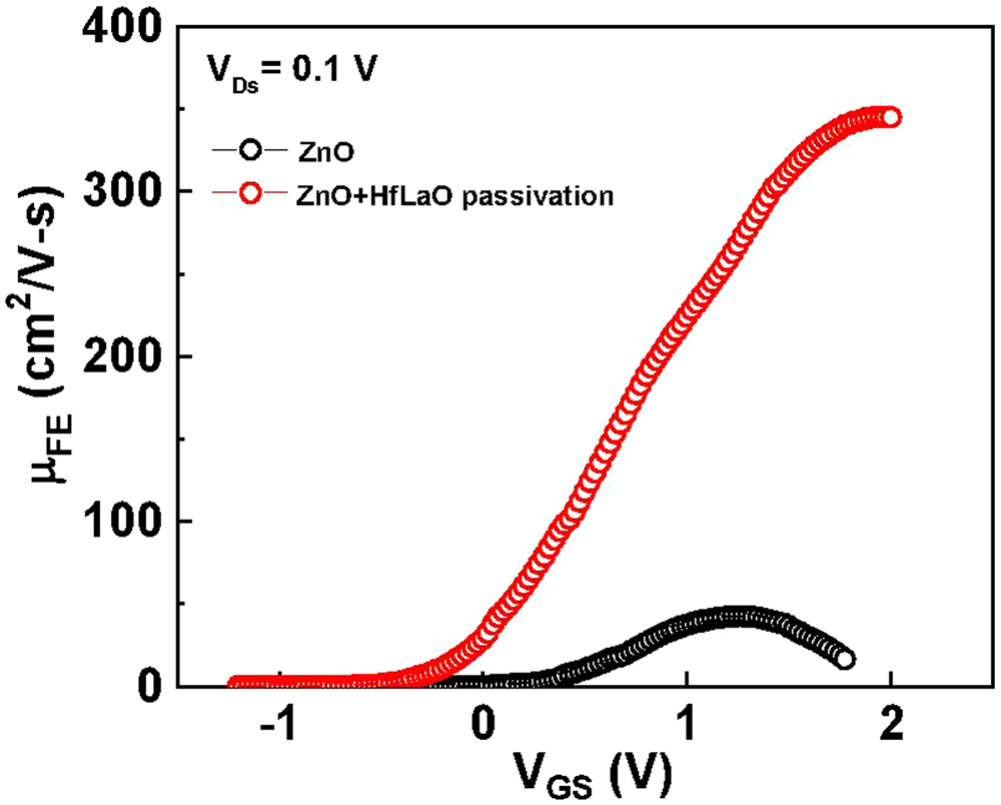
The *μ*_*FE*_-*V*_*GS*_ characteristics of ZnO/high-κ/TaN TFT on flexible PEN without and with HfLaO passivation.

The *μ*_*EF*_-*V*_*GS*_ characteristics is plotted in Fig. 3 from the measured *I*_*DS*_-*V*_*GS*_ characteristics. For control non-passivated devices, an acceptable peak *μ*_*FE*_ of 43 cm^2^/Vs is obtained for room-temperature-processed ZnO TFT. The TFT devices after HfLaO passivation has remarkably high *μ*_*FE*_ of 345 cm^2^/Vs; this is the highest value for TFT on flexible substrate^1–7^ and is even higher than the reported IGZO and ZnON TFTs fabricated on rigid substrate^22, 23^. The much improved *μ*_*FE*_ for HfLaO-passivated device is owing to the higher *I*_*ON*_ and the lower *I*_*OFF*_. It is important to notice that the *μ*_*FE*_ decreases monotonically with decreasing gate length^32, 33^:

1 $$\mu \approx {\mu }_{0}\frac{L}{L+{\mu }_{0}W{C}_{ox}{R}_{SD}({V}_{GS}-{V}_{th})}$$

where R_SD_ is the source/drain series resistance, μ_FE_ is the apparent field-effect mobility and μ_0_ is the true field effect mobility. At long gate length, the μ_FE_ is approaching to μ_0_; thus, the long 48 μm gate length device was used.

**Figure 4:**
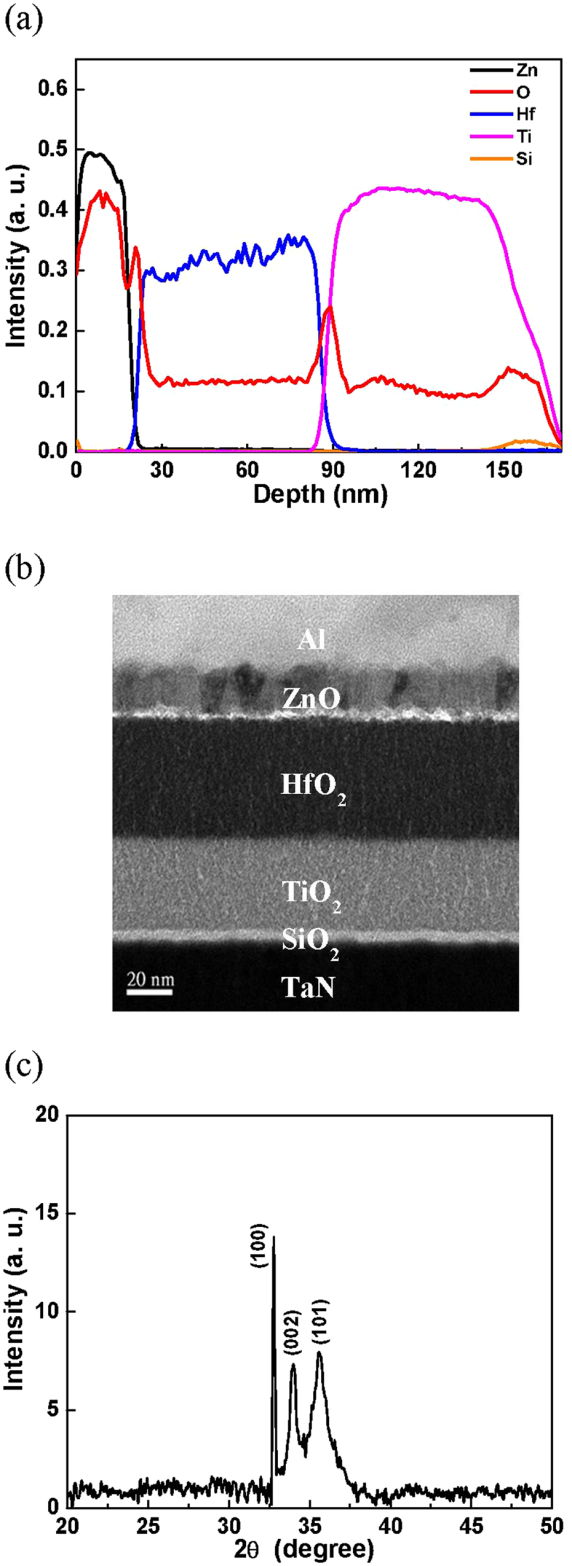
(**a**) SIMS depth profile of ZnO on HfO_2_/TiO_2_/SiO_2_ stacked gate dielectrics, (**b**) Cross-sectional TEM and (**c**) XRD spectra of ZnO/high-κ/TaN structure on flexible PEN substrate.

To understand the mechanism of such large mobility improvement, material and structure analysis were performed. Figure 4(a) shows the secondary ion mass spectrometry (SIMS) depth profile, where a ZnO channel, HfO_2_, TiO_2_, and thin SiO_2_ gate dielectric stack were recognized. The device structure of Al contact, ZnO channel, high-κ gate dielectric stack, and TaN metal-gate were also observable from the cross-sectional transmission electron microscopy (TEM) image shown in Fig. 4(b). The ZnO active layer forms columnar nano-crystalline structure with a size of ~10–20 nm. The formed crystalline structure is further evidenced from the X-ray diffraction (XRD) spectra shown in Fig. 4(c). Highly oriented phases of XRD peaks were measured, even though the ZnO was deposited by sputtering at room temperature. The full-width at half-maximum (FWHM) of XRD spectra are comparable with the data of ZnO published in literature^34, 35^, while the IGZO has an amorphous structure^36^.

**Figure 5:**
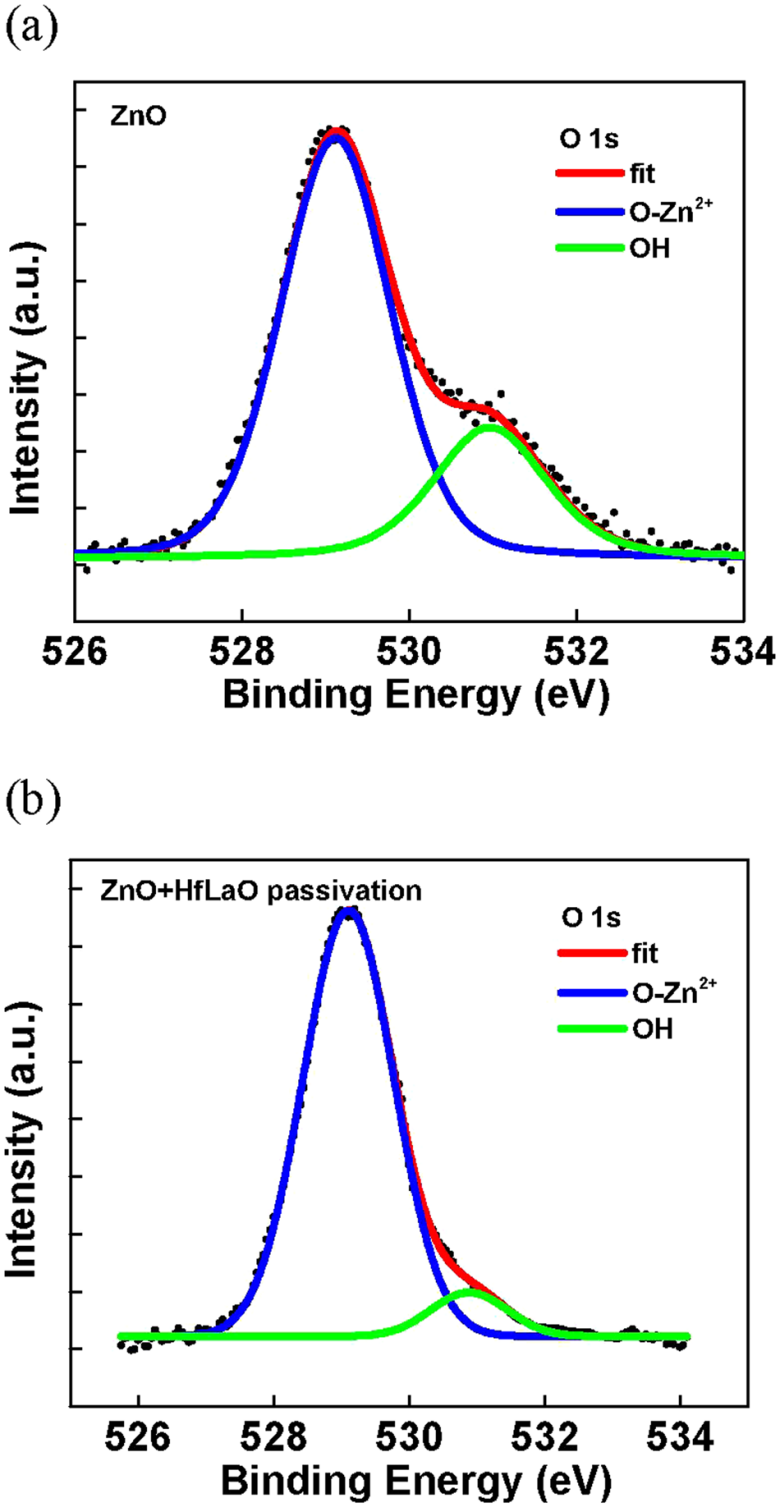
XPS spectra of ZnO/high-κ/TaN structure on flexible PEN substrate (**a**) without and (**b**) with HfLaO passivation. Here the passivated HfLaO was removed for XPS measurement.

Figure 5(a,b) show the XPS spectra without and with HfLaO passivation, respectively. The atomic composition of nano-crystalline structure is identified to be Zn^2+^-O^2−^, as measured from the XPS spectra. It is important to notice that significant amount of OH bonding signal was also measured for non-passivated device. The OH bonding in nano-crystalline ZnO was originated from the moisture absorption of ambient air, even though dry process steps were used to fabricate the devices. Similar strong moisture absorption is well known in IGZO to cause degradation.

The chemical reaction of ZnO and H_2_O is expressed as:

2 $${\rm{ZnO}}+{{\rm{H}}}_{2}{\rm{O}}\to {\rm{Zn}}{({\rm{OH}})}_{2}$$

In addition to surface, the tiny H_2_O molecule can also react with grain boundaries through the thin 20-nm ZnO. Here the grain boundaries are highly reactive due to their high defect density. Once the Zn(OH)_2_ was formed, it damaged the Zn-O bonded nano-crystal and created dangling bonds that further form charged states in the ZnO bandgap. The decreased XPS OH signal and related charged defects are also supported from the high positive charge density (ΔQ_p_) of 2 × 10^12^ cm^−2^, which was obtained from the V_T_ shift (ΔV_T_) between HfLaO-passivated and non-passivated ZnO devices shown in Fig. 3(b), from the ΔQ_p_ = C_G_ × ΔV_T_. Such positive charges and dangling bonds also found in the interim SiO_x_ region between SiO_2_ and Si body, the origin of positive fixed oxide charges in SiO_2_/Si metal-oxide-semiconductor field-effect transistor (MOSFET) shown in text book. On the other hand, the OH bonding signal in XPS spectra of Fig. 5(b) is much lowered for HfLaO passivated ZnO device. It is well known the high-κ gate dielectric will absorb the moisture^25–29^, especially the La_2_O_3_, which in turn reduce the Zn(OH)_2_ formation.

**Figure 6:**
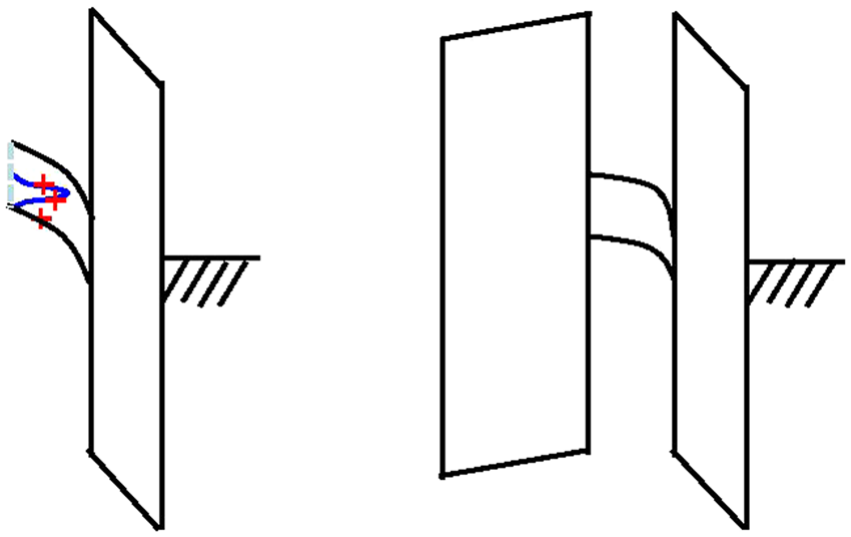
Schematic ZnO/high-κ/TaN energy bands and nano-crystalline-ZnO band structures on flexible PEN without (left) and with (right) HfLaO-passivation.

The high-density positive ΔQ_p_ further causes Fermi-level closer to valance band, increases the ZnO depletion region, and lower the n-type ZnO conduction, as shown in the schematic diagrams of Fig. 6. The electron wave-function in a MOSFET typically distributes over 20 nm^30^; therefore the high-density ΔQ_p_ will also increase electron scattering rate and decrease mobility. However, the passivation does not affect the gate EOT, because the EOT of a TFT only counts the dielectric next to the gate. Because proper passivation blocks the reaction between H_2_O and ZnO, the OH bonding in HfLaO/ZnO is much reduced to lower ΔQ_p_ and potential barriers at grain boundaries. This in succession leads to much higher mobility, because the ZnO has overlapped s-orbitals for conduction.

Table 1 compares the device performance of various materials on flexible and rigid substrates. The mobility of HfLaO-passivated ZnO TFT is higher than the IGZO and ZnON TFTs on rigid substrate^22, 23^ that is also the record highest value for TFTs on flexible substrate^1–7^. This is possible because the poly-crystalline material always has better material quality and higher mobility than amorphous structure, and the mobility improvement can be as large as ~100 times for poly-Si versus amorphous-Si TFTs. The very high mobility ZnO TFT with excellent *SS*, large *I*_*ON*_/*I*_*OFF*_ and low V_D_ are vital for both DC and AC power saving. The simple process and low material cost of nano-crystalline ZnO device should have strong impact on next generation display, as long as OH bonding related charge traps and grain boundary potential barriers are improved by proper passivation. The achieved high mobility on amorphous material is also the enabling technology for high-speed 3D brain-mimicking chip^24^.

**Table 1:** Device performance comparison among flexible ZnO, MoS_2_, and HfLaO-passivated ZnO TFTTs (this work).

Channel Materials	Channel layer thickness (nm)	Gate Insulator Materials	*SS* (V/decade)	*μ*_*EF*_ (cm^2^/V · s)	*I*_*ON*_/*I*_*OFF*_	Operating Voltage (V)
Flexible ZnO [1]	10	Al_2_O_3_	—	~32	~10^8^	8
Flexible MoS_2_ [2]	~2.6	hBN	—	~40	>10^4^	80
Rigid ITO/IGZO [22]	5/70	SiO_2_	~0.25	104	>10^7^	15
Rigid ZnON [23]	50	SiN_x_/SiO_2_	0.8	~115	>10^6^	20
Flexible This Work	20	HfO_2_/TiO_2_/SiO_2_	0.103	345	7 × 10^6^	2

The IGZO and ZnON TFTs on rigid glass substrate were also added for comparison.

In conclusion, very high mobility, excellent *SS*, large *I*_*ON*_/*I*_*OFF*_, low V_D_, and low power operation were achieved in ZnO TFT device that is crucial for display and 3D IC. The excellent device integrity is due to the novel passivation scheme with simple process.

## Methods

The bottom-gate ZnO/high-κ/metal-gate TFTs were made on flexible polyethylene naphthalate (PEN) substrate. In addition to its low cost, the PEN substrate has good properties of a low linear thermal expansion coefficient, surface smoothness, and optical clarity. A 300-nm smoothing SiO_2_ layer was first deposited on PEN substrate. Then the 60-nm TaN gate metal, tri-layer gate dielectrics of 50-nm HfO_2_, 40-nm TiO_2_ and 4-nm-SiO_2_, and a 20-nm ZnO active layer were deposited by physical vapor deposition (PVD). Then the Al source/drain (S/D) electrodes was formed. Finally, the device was passivated by 20 nm thick HfLaO dielectric with opened S/D probing window. The TaN gate electrode was deposited by sputtering at a power of 800 W, Ar/N_2_ of 100/10 sccm, and a pressure of 3 × 10^−3^ torr. The gate dielectric stacks were deposited by electron-gun evaporation at 5 KV, and the deposition rates were 0.24/0.2/0.1 Å/sec, respectively. The ZnO channel were deposited by sputtering at a power of 100 W, Ar/O_2_ of 20/5 sccm, and 1 Å/sec deposition rate. The Al source-drain was deposited by thermal evaporation deposition. The HfLaO was deposited by electron-gun evaporation at a deposition rate of 0.15 Å/sec. Before deposition, the chambers were pumped down to 3 × 10^−6^ torr. The low deposition rate is important to reach good quality. No post-deposition annealing was used that is the merit of this work. The gate size of fabricated TFT is 48-μm × 505-μm. To investigate the large mobility improvement, X-ray diffraction (XRD), secondary ion mass spectrometry (SIMS), cross-sectional transmission electron microscopy (TEM), and X-ray photoelectron spectroscopy (XPS) were used to analyze the material property. Very low etching rate of 0.2 Å/sec was used in the XPS measurement due to the thin 20 nm HfLaO passivation layer.

## Electronic supplementary material

Supplementary information
