# Supplementary material for: Remarkably High Mobility Thin-Film Transistor on Flexible Substrate by Novel Passivation Material
Source: Sci Rep. 2017 Apr 25;7:1147. doi: 10.1038/s41598-017-01231-3 (PMC5430887; doi:10.1038/s41598-017-01231-3)
Supplement: Supplementary file 1 — Supplementary information [file 41598_2017_1231_MOESM1_ESM.pdf]

Remarkably High Mobility Thin-Film Transistor on Flexible Substrate by Novel Passivation Material

Cheng Wei Shih, Albert Chin*

Department of Electronics Engineering, National Chiao Tung University, Hsinchu 300, Taiwan

*Correspondence: Albert Chin (email: achin@faculty.nctu.edu.tw)

Supplementary Figures

(a)

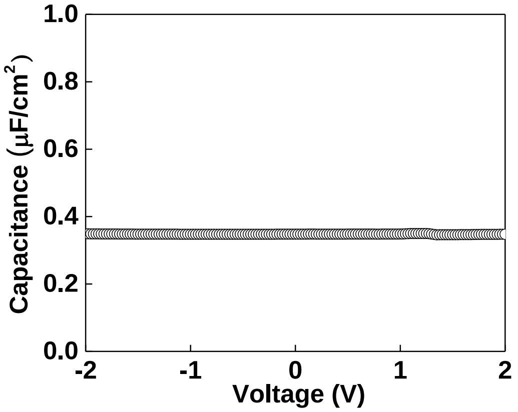

(b)

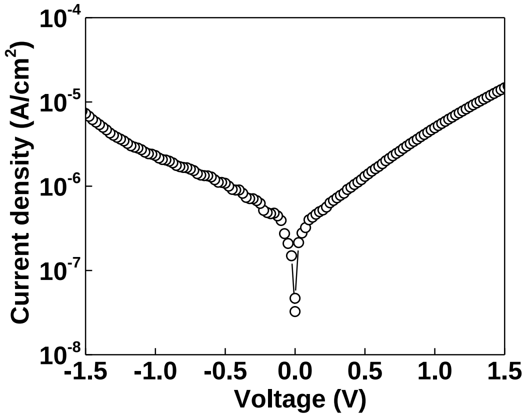

Figure S1. (a) *C-V* and (b) *J-V* characteristics of Al/high-κ/TaN capacitor on flexible PEN.
